# Correction: Construction of a prognostic model for colorectal cancer liver metastasis based on single-cell transcriptomics and regulation of the MIF pathway

**DOI:** 10.3389/fonc.2025.1731870

**Published:** 2025-12-15

**Authors:** Jianqiang Cao, Zhaobin He, HuiJie Gao, Yongzhe Yu, Shengbiao Yang, Xiqiang Wang, Jun Niu, Cheng Peng

**Affiliations:** 1 Department of Hepatobiliary Surgery, General Surgery, Qilu Hospital, Cheeloo College of Medicine, Shandong University, Jinan, China; 2 Department of Geriatrics, Qilu Hospital, Shandong University, Jinan, China

**Keywords:** single-cell transcriptomics, colorectal cancer, liver metastasis, targeted therapy, prognostic model

An incorrect **Funding** statement was provided. The correct **Funding** statement reads:

“The author(s) declare financial support was received for the research and/or publication of this article. The National Natural Science Foundation of China grant number 82072674 and The Natural Science Foundation of Shandong Province grant number ZR2020MH258 funded this research.”

The original version of this article has been updated.

