# Supplementary figures and images for: Construction of a prognostic model for colorectal cancer liver metastasis based on single-cell transcriptomics and regulation of the MIF pathway
Source: Front Oncol. 2025 Oct 7;15:1588514. doi: 10.3389/fonc.2025.1588514 (PMC12537406; doi:10.3389/fonc.2025.1588514)

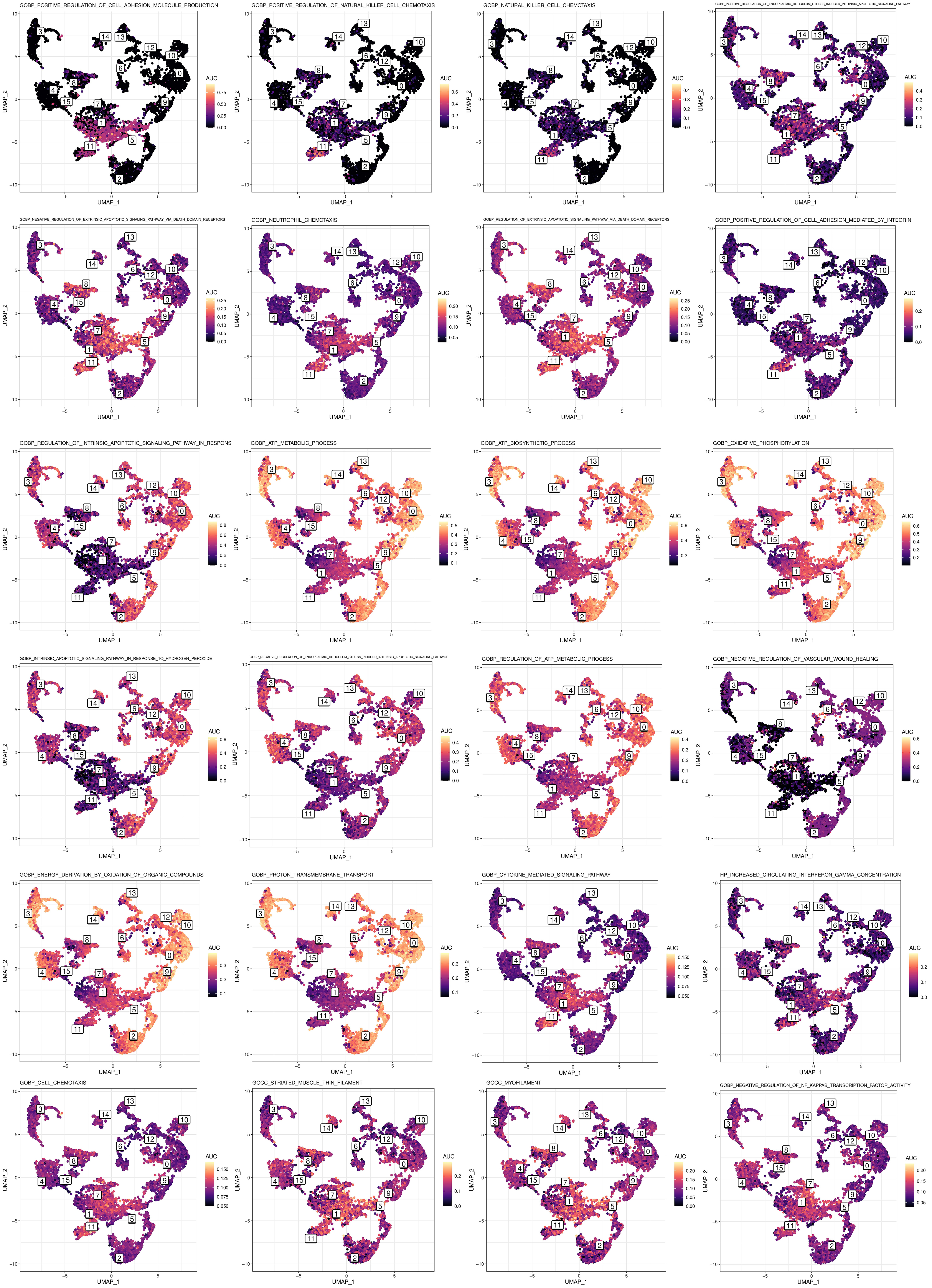

Supplement: Supplementary file 5 [file Image2.jpeg]

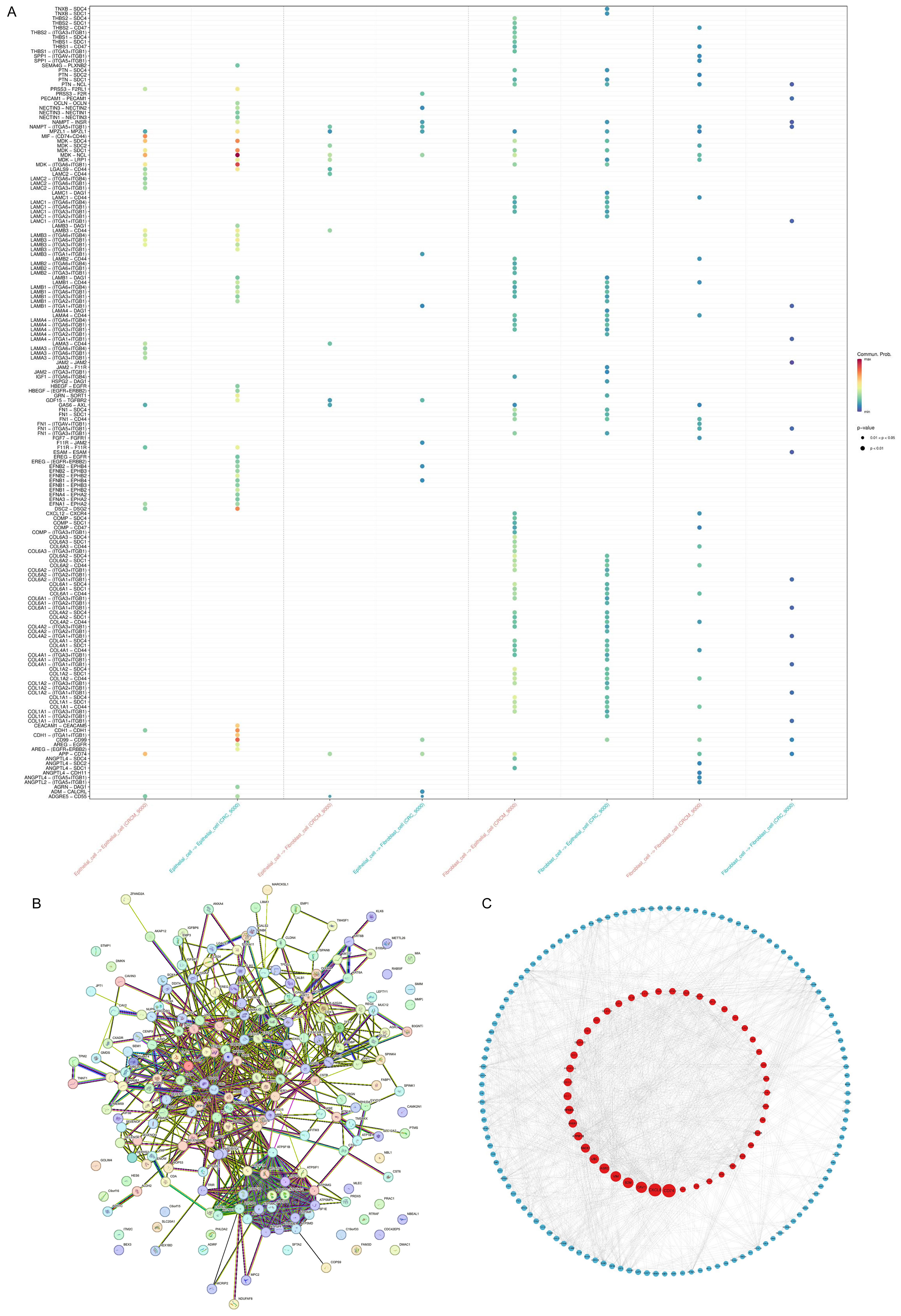

Supplement: Supplementary file 8 [file Image5.jpeg]
